# Differential Gene Profiling of the Heartwood Formation Process in *Taiwania cryptomerioides* Hayata Xylem Tissues

**DOI:** 10.3390/ijms21030960

**Published:** 2020-01-31

**Authors:** Ting-Feng Yeh, Jui-Hua Chu, Li-Yuan Liu, Shih-Yin Chen

**Affiliations:** 1 School of Forestry and Resource Conservation, National Taiwan University, Taipei 10617, Taiwan; r01625002@ntu.edu.tw (L.-Y.L.);; 2 Center for Systems Biology, National Taiwan University, Taipei 10617, Taiwan

**Keywords:** differentially expressed genes, extractives, heartwood formation, ray parenchyma cell, *Taiwania cryptomerioides* Hayata, transition zone

## Abstract

Taiwania (*Taiwania cryptomerioides*) is an important tree species in Taiwan because of the excellent properties of its wood and fascinating color qualities of its heartwood (HW), as well as the bioactive compounds therein. However, limited information is available as to the HW formation of this species. The objective of this research is to analyze the differentially expressed genes (DEGs) during the HW formation process from specific Taiwania xylem tissues, and to obtain genes that might be closely associated with this process. The results indicated that our analyses have captured DEGs representative to the HW formation process of Taiwania. DEGs related to the terpenoid biosynthesis pathway were all up-regulated in the transition zone (TZ) to support the biosynthesis and accumulation of terpenoids. Many DEGs related to lignin biosynthesis, and two DEGs related to pinoresinol reductase (PrR)/pinoresinol lariciresinol reductase (PLR), were up-regulated in TZ. These DEGs together are likely involved in providing the precursors for the subsequent lignan biosynthesis. Several transcription factor-, nuclease-, and protease-encoding DEGs were also highly expressed in TZ, and these DEGs might be involved in the regulation of secondary metabolite biosynthesis and the autolysis of the cellular components of ray parenchyma cells in TZ. These results provide further insights into the process of HW formation in Taiwania.

## 1. Introduction

Wood formation in trees develops from five major steps, including cell division, cell expansion, cell wall thickening, programmed cell death, and heartwood (HW) formation [1]. The first four steps proceed from several cell layers around the cambium region of a tree stem, whereas HW formation usually occurs a couple of annual rings away from the cambium region. Hence, HW is normally defined as the inner layers of wood without living cells, and the reserve materials (e.g., starch) turn into HW substance in the cells, whereas sapwood (SW) has both living cells and the reserve materials [2,3]. In between the SW and HW, the transition zone (TZ) is located. The HW formation process may accumulate substantial amounts of secondary metabolites (extractives) into cell lumens or tissues. This process normally produces inner darker-colored HW, often easily recognizable from the paler outer SW. These extractives often render HW its natural durability, esthetic characteristics, pharmaceutical applications, and commercial value [3].

Taiwanina (*Taiwania cryptomerioides* Hayata) is a monotypic genus species, and together with Sequoia and Metasequoia, is considered as a living fossil dating back to the Tertiary period about 65 to 1.806 million years ago [4]. Taiwania is one of the most economically important plantation species in Taiwan and its HW signature is yellowish-red with purplish-pink streaks. The wood properties of Taiwania are known for excellent decay resistance and durability when used in harsh environments [5]. The extractives accumulated in the cell lumens of the HW region provide the superior durability of Taiwania wood [6,7]. There are more than 500 compounds that have been isolated from Taiwania, and these compounds include lignans, diterpenoids, sesquiterpenoids, flavonoids, cyclitols, and sterols [8,9]. In Taiwania’s HW extractives, lignans and terpenoids are the two major components, and many of these compounds have proven to have outstanding performances in antifungal [10], antitermite [6], antibacterial [11], antitumor [12,13], and anti-inflammatory [14] activities. Although these compounds have been demonstrated to have superior potential in bioactivities, information on how these bioactive compounds accumulate into HW in Taiwania is very limited.

HW formation is described as a form of programmed cell death [15], and it has been suggested that the longevity of the ray parenchyma cells and the depletion of reserve materials are closely related to the HW formation process [3,16]. Previous research has shown that the ray parenchyma cells of Taiwania gradually lose the starch grains from the inner SW and lose their nuclei in TZ, whereas the colored substances deposit into the cell lumens in the TZ [17]. Overall, the ray parenchyma cells lose their vitality within 1–2 annual rings in the TZ, which would appear between 8-13 year rings counted from the cambium, depending on individual trees [17]. Similar results were also reported for other species, and the occurrence of this TZ is species-dependent, with the year ring numbers from cambium being 7-8 in *Larix kaempferi* [18], 12–23 in *Cryptomeria japonica* [19], and 14-22 and 28-33 in *Pinus rigida* and *Pinus densiflora* [20]. The extractives often start to accumulate from inner SW, increase in TZ, and deposit to a high level in the HW region [17,20,21].

It seems that the HW formation process from the SW transition to HW is characterized by a shift of cell vitality and temporary activation leading to the biosynthesis of HW extractives. In *Robinia pseudoacacia* (black locust) and *Juglans nigra* (black walnut), the accumulations of flavonoids in TZ correlated with the transcript levels or protein activities of phenylalanine ammonia lyase (PAL), chalcone synthase, flavanone 3-hydroxylase, and dihydroflavonol 4-reductase genes [22,23,24,25], supporting active flavonoid biosynthesis in TZ. A proteomics study on protein extracts from SW and TZ of black locust further indicated that the proteins responsible for carbohydrate metabolism and flavonoid turnover were highly expressed in SW, whereas those for flavonoid biosynthesis were strongly expressed in TZ [26]. A recent study on HW formation in *Pinus sylverstris* (Scots pine) also indicated that transcripts related to phenylpropanoid and stilbene biosynthesis were elevated, leading to stilbene biosynthesis in TZ [27]. A unique expression pattern of the transcripts related to sesquiterpenoid biosynthesis was reported to be preferentially located in the HW of *Santalum album* (sandalwood) [28]. All evidence suggests that HW formation seems to be an actively regulated and continuous stage of woody plant development. Despite the significance of these gene and enzyme discoveries on HW formation from the above species, however, information related to the molecular mechanisms underlying the dynamic process of the accumulation of bioactive compounds in HW formation in Taiwania is limited. This hinders the understanding of the unique aspects of Taiwania’s wood formation process.

The aim of this study is to carry out RNA-sequencing (RNA-seq) experiments from specific xylem tissues representing the HW formation process of Taiwania, and to identify genes that might be associated with this process. The results show that differentially expressed genes (DEGs) related to terpenoid, phenylpropanoid, and lignan biosynthesis pathways are up-regulated in TZ to support the biosynthesis and accumulation of terpenoids and lignans into Taiwania HW. Several DEGs encoding transcription factors, nucleases, and proteases are highly expressed in TZ, supporting secondary metabolite biosynthesis and autolysis of the cellular components of ray parenchyma cells in Taiwania’s HW formation process.

## 2. Results and Discussion

### 2.1. Sampling of Wood Tissues Containing Living Ray Parenchyma Cells

**Figure 1:**
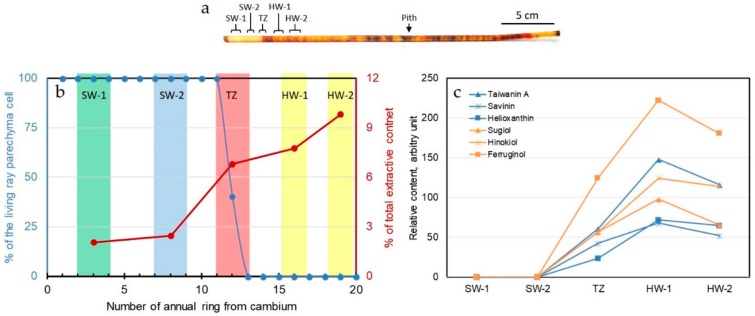
Localization of different tissue segments used for total RNA extraction and extractive content analysis of Tree A. (**a**) The increment core showing characteristic orange-purple heartwood and pale-yellow sapwood; (**b**) Distribution of living ray parenchyma cells and total extractive content; (**c**) The distribution of Taiwania’s signature bioactive compounds in different tissues. SW-1, outer sapwood; SW-2, inner sapwood; TZ, transition zone; HW-1, outer heartwood; HW-2, inner heartwood.

It has been suggested that active living ray parenchyma cells are related to the HW formation process in many species [16,20,24,26]. In order to obtain living cells containing vital transcript information in relation to the HW formation process, thin radial sections were obtained and observed under a microscope along the increment cores (Figure 1a). The percentages of the living ray parenchyma cells were counted ring by ring along the radial direction of the cores. A total of three trees (Tree A, B, and C) were used in this study. Using Tree A as the example, the results (Figure 1b) indicate that the living ray parenchyma cells were distributed from outer SW (SW-1) and inner SW (SW-2), and quickly ceased in TZ. No living ray parenchyma cell was found in the HW region (HW-1 and HW-2). Hence, that the annual rings in Taiwania have a transition from 100% living ray parenchyma cells to 0% living ray parenchyma cells is defined as the TZ in this study. As for the radial distribution of extractives (Figure 1b), the total extractive contents gradually increased from SW-1 to SW-2, further increased in TZ, and became even higher toward the HW regions (HW-1 and HW-2). Meanwhile, the distributions of the characteristic bioactive compounds such as lignans (taiwanin A, savinin, and helioxanthin) and diterpenoids (sugiol, hinokiol, and ferruginol), were found distributed in TZ, HW-1, and HW-2 (Figure 1c). The existence of these compounds in SW regions is below our instrument’s detection limit. Overall, for all three trees, the living ray parenchyma cells were only distributed in SW (SW-1, SW-2) and TZ, and this phenomenon was accompanied with the gradual accumulation of extractives and bioactive compounds from TZ toward HW region. Hence, only tissue segments from SW-1, SW-2, and TZ were used for the total RNA extractions, and these RNAs were submitted to the subsequent RNA-seq study. No tracheid cells were alive in these SWs and TZ, and Taiwania is known for having no resin canal and having sparse longitudinal parenchyma cells [29]. Thus, the RNA isolated from these tissues should represent the RNA majority from living or deceasing ray parenchyma cells.

### 2.2. Transcriptome Assembly and Functional Annotation

Illumina RNA-seq technology was used to sequence the woody tissues representing the HW formation process of Taiwania trees. A total of 9 RNA-seq libraries, 3 tissues (SW-1, SW-2, and TZ) per tree for 3 trees (Tree A, B, and C), were sequenced. After removing sequencing adapters, as well as low quality and ambiguous reads, the final assembly of the transcriptome library representing Taiwania HW formation had 56,092 contigs with an N50 length of 1,432 bp. To reduce the redundancy of assembled contigs, the longest transcripts were retained as the unigenes. The longest transcript collection has 36,654 unigenes with an N50 of 1,209 bp (Table S1). These 36,654 unigenes were further BlastX-searched against NCBI non-redundant, Swiss-Prot, and *Arabidopsis thaliana* protein sequences (TAIR 10.1) with an E-value threshold of 1 × 10^−5^. Gene ontology (GO) annotation was performed using the Blast2GO program (Version 4.1.9) [30]. Functional GO analysis of this longest unigene collection revealed that in the category of cellular components (Figure S1), cell (GO:0005623), cell part (GO:0044464), and organelle (GO:0043226) are the most noticeable. In the category of molecular function, the majority of these genes were related to catalytic activity (GO:0003824) and binding (GO:0005488). For the category of biological process, the most prominent GO terms were related to cellular process (GO:0009987), metabolic process (GO:0008152), and single-organism process (GO:0044699), indicating that these unigenes were involved in several metabolic activities related to the HW formation process.

### 2.3. Analysis of the Differentially Expressed Genes (DEGs) in Different Tissues

**Figure 2:**
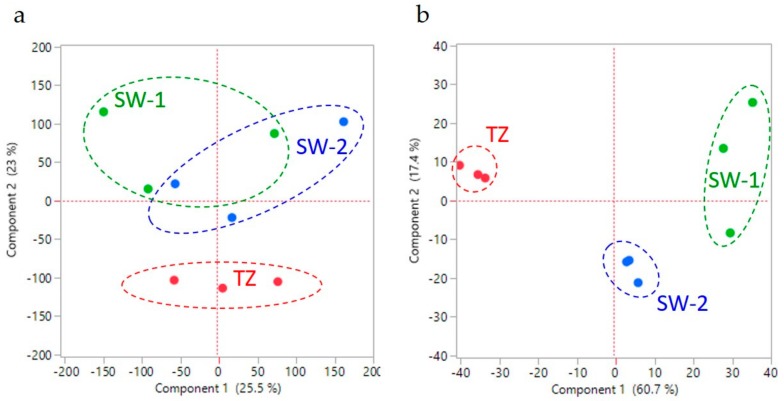
Principle component analysis of the normalized gene expression level (reads per kilobase per million mapped reads) using (**a**) longest unigenes, and (**b**) differentially expressed genes. SW-1, outer sapwood; SW-2, inner sapwood; TZ, transition zone.

Based on three pairwise comparisons (SW-1 vs. SW-2; SW-2 vs. TZ; and SW-1 vs. TZ), a total of 1428 DEGs were identified under the thresholds of log_2_ (fold change) over 1, and corrected false discovery rate (FDR) *p*-values of less than 0.005 (Table S2). The global expression differences and similarities between these three tissues were first analyzed using principal component analysis (PCA). Using the normalized gene expression levels (reads per kilobase per million mapped reads, RPKM) of all longest unigenes, Component 1 (25.5%) and Component 2 (23%) together could only explain a total of 48.5% of the variation between SW-1, SW-2, and TZ (Figure 2a). However, using the normalized gene expression levels (RPKM) of DEGs (Figure 2b, Table S3) clearly better resolves separation of these three tissues. Component 1 (60.7%) and Component 2 (17.4%) together explain about 78.1% of the variance of these three tissues, indicating that the current DEG separation criterion has captured the major information from this HW formation process of these tree tissues.

**Figure 3:**
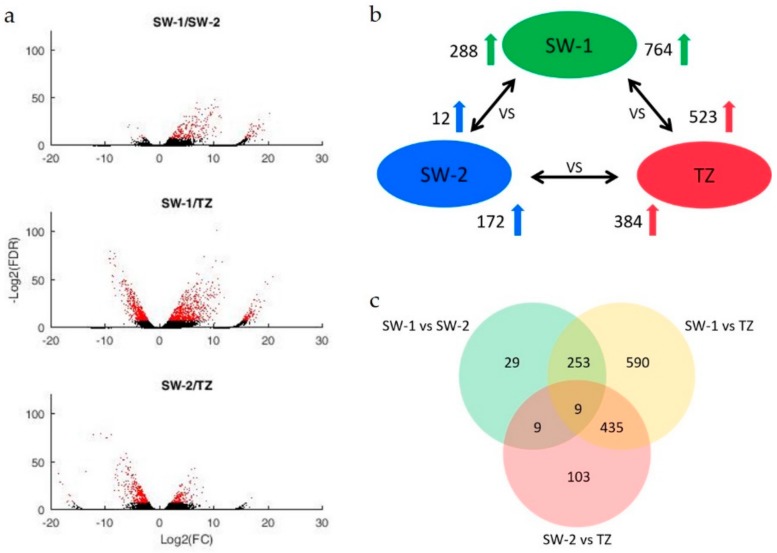
DEGs log_2_ (fold change) > 1; FDR *p*-value < 0.005) between any two different tissues of the HW formation process. (**a**) Volcano plots between two tissues, DEGs in red dots; (**b**) The numbers of significantly up-regulated genes between any two tissues (double-headed arrows); (**c**) Common and unique DEGs between any comparison groups represented in Venn diagram. SW-1, outer sapwood; SW-2, inner sapwood; TZ, transition zone, DEG, differentially expressed gene; FDR, false discovery rate.

A further survey of these 1428 DEGs indicated that 288 and 12 DEGs were differentially up-regulated in SW-1 and SW-2 tissues when comparing SW-1 and SW-2 (Figure 3a,b, Table S2). 764 and 523 DEGs were differentially up-regulated in SW-1 and TZ tissues when comparing SW-1 and TZ tissues (Figure 3a,b, Table S2). 172 and 384 DEGs were differentially up-regulated in SW-2 and TZ tissues when comparing SW-2 and TZ tissues (Figure 3a,b, Table S2). The number of DEGs apparently changed during the HW formation process. The DEGs from the SW-1 vs. TZ comparison represented the majority (1287 DEGs) of the 1428 DEGs from these three tissues (Figure 3c, Table S2), indicating that there are dramatically different biological events between SW-1 and TZ tissues. Of these (1287 DEGs), 590 DEGs are unique to SW-1 vs. TZ, 253 and 435 DEGs are common to SW-1 vs. SW-2 and SW-2 vs. TZ, and 9 DEGs are common to all three comparisons.

Based on these, the GO enrichment analysis (Fisher’s exact test) was performed focusing on all these DEGs. The significant level was set with corrected FDR *p*-value at 0.05. GO enrichment analyses were carried for biological process (Figure S2), molecular function (Figure S3), and cellular component (Figure S4). GO terms related to secondary metabolic process (GO:0019748), carbohydrate metabolic process (GO:0005975), cell wall organization or biogenesis (GO:0071554), response to stress (GO:0006950), transmembrane transporter activity (GO:0022857), oxidoreductase activity (GO:0016491), hydrolase activity (GO:0016798, acting on glycosyl bonds), ion biding (GO:0043167), cell wall (GO:0005618), etc., were typically enriched in DEGs from the three GO categories. This result indicated that these functions or processes were involved in the HW formation process in Taiwania.

The DEGs were further referenced through the KEGG database. A total of 312 DEGs can be assigned into 88 pathways and 168 EC numbers (Table S4). The assigned major pathways were involved in purine metabolism (18.2% of assigned DEGs), biosynthesis of antibiotics (15.7% of assigned DEGs), thiamine metabolism (13.8% of assigned DEGs), starch and sucrose metabolism (13.5% of assigned DEGs), phenylpropanoid biosynthesis (11.5% of assigned DEGs), flavonoid biosynthesis (9.0% of assigned DEGs), pentose and glucoronate interconversion (7.0% of assigned DEGs), terpenoid backbone biosynthesis (5.4% of assigned DEGs), and fatty acid degradation (5.4% of assigned DEGs). These results are consistent with our findings (Figure 1c) that the extractives, lignans and terpenoids, were accumulated from the SW toward TZ in Taiwania stems.

**Figure 4:**
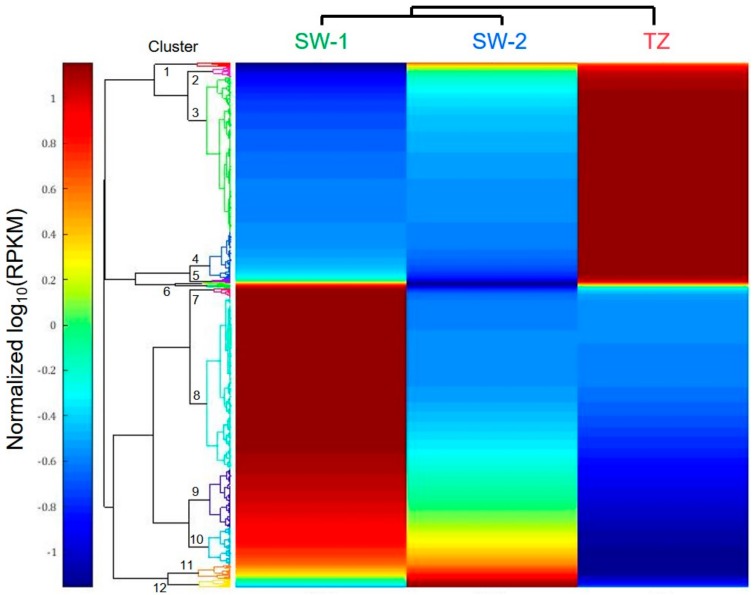
Hierarchical cluster analysis of the 1428 DEGs with differential expression across three different tissues. SW-1, outer sapwood; SW-2, inner sapwood; TZ, transition zone. The log_10_ (RPKM) value of the same DEG was normalized between 1 and -1 within three tissues. RPKM, reads per kilobase per million mapped reads.

Hierarchical clustering (Euclidean distance < 0.8) was applied to further analyze the expression profiles of these 1428 DEGs over three tissues. The results showed that the gene expression profiles between SW-1 and SW-2 were more closely related when compared to that of TZ (Figure 4). These DEGs expression profiles can be majorly grouped into 2 groups and 12 clusters (Figure 4, Table S5). Group 1 contains Clusters 1 to 6, which have generally lower expression levels in the SW regions and higher expression levels in TZ. DEGs’ functions related to some phenylpropanoid, terpenoid, and flavonoid biosynthesis are in this group. DEGs with functions of starch-degrading enzymes and nucleases are also harbored in Group 1. Phenylpropanoid and terpenoid biosynthesis-related genes were expressed more toward TZ, and this might be related to the accumulation of bioactive compounds found in Figure 1. Similar results were reported in conifer and hardwood species, such as *P. sylvestris* (Scots pine), *J. nigra* (black walnut), and *R. pseudoacacia* (black locust), that the transcription levels of genes related to the biosynthesis of phenolic or flavonoid compounds were correlated with the accumulation of HW extractives in the TZ of these species [22,25,27]. In Taiwania, the starch grains were deposited in the ray parenchyma cells of SW-2, and the ray parenchyma cells would gradually lose their nuclei and starch grains in the TZ region, where the deposition of colored extractives started [17]. Higher-expressed starch-degrading genes are likely related to the degradation of starch to provide the carbon source for the biosynthesis of the bioactive compounds, and the expression of nuclease-encoding genes toward TZ may be related to the degradation of nuclei in the ray parenchyma cells. On the other hand, Group 2 contains Clusters 7 to 12, which have generally lower expression levels in the TZ region and higher expression levels in SW regions (Figure 4, Table S5). With regard to DEGs’ functions related to cell wall polysaccharides (cellulose, xylan, mannan, galactan, etc.) biosynthesis-related enzymes are particularly implicated in this group. Research in HW formation of black locust also indicated that genes related to cell wall synthesis were more expressed in SW compared to that in TZ [25]. Higher expressions of cell wall biosynthesis-related genes toward SW regions are related to more cell wall biosynthesis activities in SW regions compared to TZ.

### 2.4. Analysis of Specific Gene Groups

It is known that diterpenoids and lignans are the dominant compounds in Taiwania’s HW, and many of them possess superior bioactivities [8,12]. Hence, we focused on terpenoid and lignan biosynthesis (secondary metabolism) pathways, and the interests were also extended to other processes, such as plant hormone signaling, transcription factors, and programmed cell death.

#### 2.4.1. Terpenoid Biosynthesis during HW Formation

**Figure 5:**
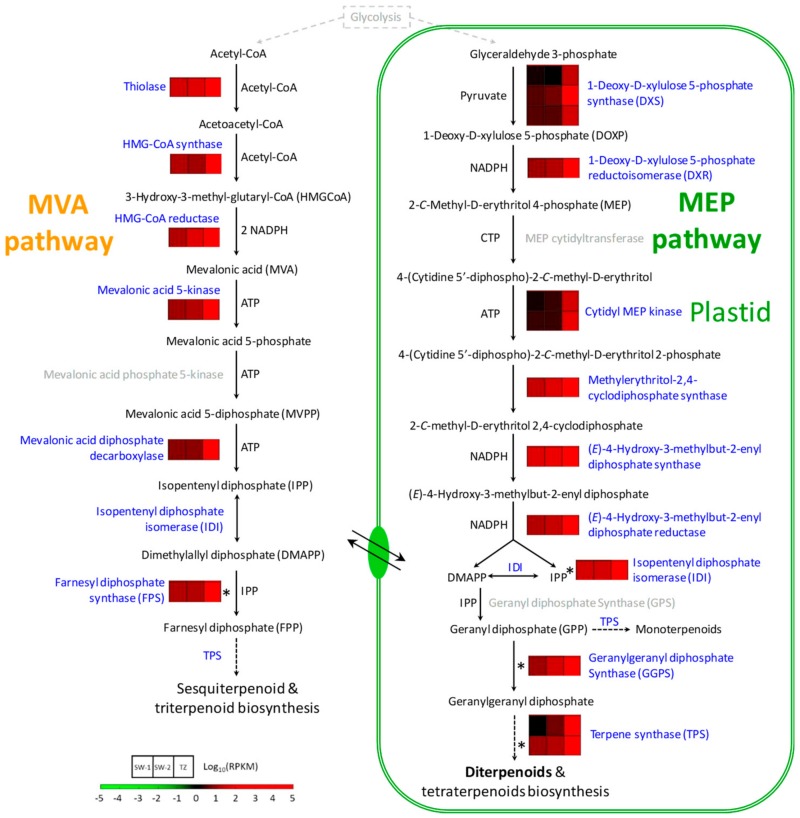
Schematic diagram of terpenoid biosynthesis pathways activated during the HW formation process in Taiwania. Each row represents the normalized gene expression level of individual DEGs, and each column represents the expression level from different tissues. SW-1, outer sapwood; SW-2, inner sapwood; TZ, transition zone. *, DEGs validated with qRT-PCR. Enzymes with no DEGs to be associated with are in gray.

The extractive contents of Taiwania gradually increased from SW-1 and SW-2 toward TZ, and the signature diterpenoids (sugiol, hinokiol, and ferruginol) were detected in TZ and further increased toward HW tissues (Figure 1c). The biosynthesis of terpenoids can be divided into four stages: (1) the synthesis of the C5 isoprene unit, (2) repetitive condensation of the C5 units to form a series of prenyl diphosphates with increasing molecular sizes, (3) conversion of prenyl diphosphates to the basic terpenoid skeletons, and (4) further modifications to the basic skeletons, such as oxidation, reduction, isomerization, and other transformations [31]. The basic C5 unit is represented by isopentenyl diphosphate (IPP) and dimethylallyl diphosphate (DMAPP). These intermediates are biosynthesized in plants from two completely different routes that are spatially separated but exchange intermediates: the mevalonate pathway (MVA pathway, Figure 5) in the cytosol, endoplasmic reticulum, and mitochondria, and the methylerythritol 4-phosphate pathway (MEP pathway, Figure 5) in the plastid. Genes and enzymes in the MVA pathway ultimately lead to the biosynthesis of sesquiterpenoids, triterpenoids, sterols, dolichols, etc., whereas those in the MEP pathway ultimately lead to the biosynthesis of monoterpenoids, diterpenoids, side-chain of chlorophyll, carotenoids, etc. [31,32].

**Figure 6:**
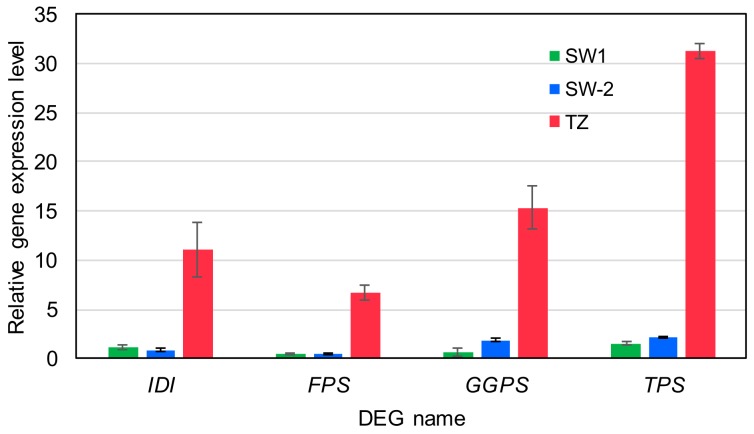
qRT-PCR validation of specific DEGs related to terpenoid biosynthetic pathway. IDI, isopentenyl diphosphate isomerase; FPS, farnesyl diphosphate synthase; GGPS, geranylgeranyl diphosphate synthase; TPS, terpene synthase. SW-1, outer sapwood; SW-2, inner sapwood; TZ, transition zone. Mean ± SD (n = 3).

DEGs related to the MEP pathway’s enzymes (Figure 5; Table S6), from 1-deoxy-D-xylulose 5-phosphate synthase (DXS) all the way to geranylgeranyl diphosphate synthase (GGPS), were up-regulated about 9- to 352-fold in TZ compared to that in SW-1 or SW-2. This indicated that more basic C5 units, IPP and DMAPP, might be provided down-stream in the pathway for biosynthesis. Two terpene synthase (TPS)-encoding DEGs were also up-regulated about 22- to 415-fold in response to the biosynthesis of the diterpenoids and other secondary metabolites. Similarly, DEGs related to the MVA pathway’s enzymes (Figure 5; Table S6), from thiolase all the way to farnesyl diphosphate synthase (FPS), were also up-regulated from SW-1 and SW-2 toward TZ. Because the MVA pathway also includes isopentenyl diphosphate isomerase (IDI) and TPSs, we cannot rule out the possibility that DEGs annotated as IDI and TPSs previously included in the plastid-localized MEP pathway might also involve in the non-plastid localized MVA pathway. Hence, the true subcellular localizations of these annotated DEGs (IDI and TPSs) would require further experimental confirmation. Overall, the DEGs of the MVA pathway were up-regulated about 10- to 106-fold in TZ compared SW-1 or SW-2. Again, this also indicated that more basic C5 units might be provided for down-stream biosynthesis of sesquiterpenoids and other compounds. Four DEGs from these two pathways were validated by quantitative reverse transcription polymerase chain reaction (qRT-PCR). The results (Figure 6) indicated that DEGs related to IDI, FPS, GGPS and TPS were highly expressed in TZ compared to SW tissues. Taken together, all of these highly expressed DEGs from both pathways in TZ contributed to the gradual accumulation of the terpenoids from TZ toward HW tissues in Taiwania stems. However unlike Taiwania, research in sandalwood (*Santalum album*), a hardwood species, has revealed that transcripts related to the MEP pathway were preferentially expressed in SW, whereas transcripts related to the MVA pathway, FPS and a subset of TPS responsible for sesquiterpenoid biosynthesis, were preferentially expressed in HW [28]. Unique living ray parenchyma cells in HW were implied to be involved in the sesquiterpenoid biosynthesis in sandalwood [16]. In the case of Taiwania, there are no living ray parenchyma cells distributed in HW region (Figure 1b), and hence, this reflects the diverse and complex terpenoid biosynthesis in HW formation from different tree species.

#### 2.4.2. Lignan Biosynthesis during HW Formation

**Figure 7:**
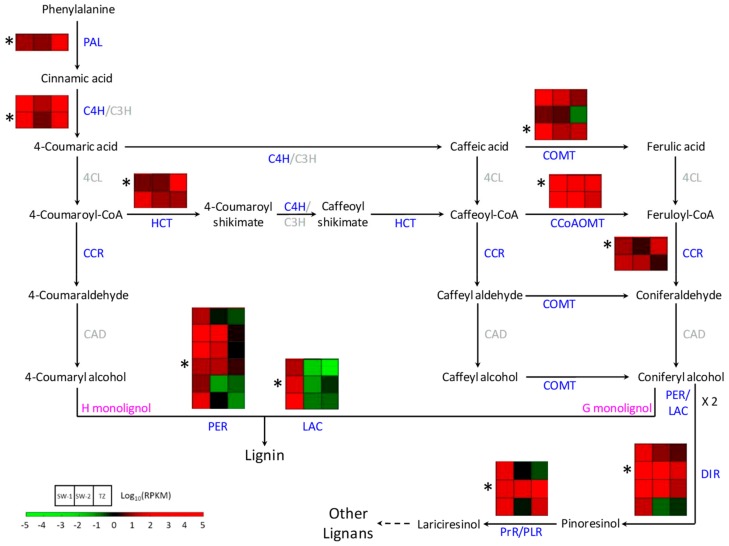
Schematic diagram of lignin and lignan biosynthesis pathways activated during the HW formation process in Taiwania. Each row represents the normalized gene expression level of individual DEGs, and each column represents the expression level from different tissues. SW-1, outer sapwood; SW-2, inner sapwood; TZ, transition zone. *, DEGs validated with qRT-PCR. PAL, phenylalanine ammonia-lyase; C4H/C3H, protein complex; C4H, cinnamate 4-hydroxylase; C3H, 4-coumarate 3-hydroxylase; HCT, 4-hydroxycinnamoyl-CoA shikimate/quinate 4-hydroxycinnamoyl transferase; 4CL, 4-coumarate CoA ligase; CCoAOMT, caffeoyl-CoA O-methyltransferase; CCR, cinnamoyl-CoA reductase; COMT, caffeic acid O-methyltransferase; CAD, cinnamyl alcohol dehydrogenase; PER, peroxidase; LAC, laccase; DIR, dirigent protein; PrR/PLR, pinoresinol reductase/pinoresinol lariciresinol reductase. Enzymes with no DEGs to be associated with are in gray.

Taiwania’s signature lignans (taiwanin A, savinin, and helioxanthin) accumulated from TZ and increased toward HW (Figure 1c). The precursor for lignan biosynthesis is coniferyl alcohol, which is also the G-monolignol for lignin biosynthesis. The supply of coniferyl alcohol starts from phenylalanine, with the aid of many enzymes in monolignol biosynthesis pathway (Figure 7). Lignin biosynthesis should be most active in tracheids close to the cambium zone in Taiwania. In the TZ, which is far away from the cambium zone, most of the lignin biosynthesis should be arrested, although some minor lignin biosynthesis activities might remain for the ray parenchyma cells. However, many of the DEGs related to monolignol biosynthesis pathways were up-regulated in TZ compared to that in either SW-1 or SW-2 (Figure 7; Table S6). These include DEGs encoded with phenylalanine ammonia-lyase (PAL), cinnamate 4-hydroxylase (C4H), 4-hydroxycinnamoyl-CoA shikimate/quinate 4-hydroxycinnamoyl transferases (HCTs), one caffeoyl-CoA O-methyltransferase (CCoAOMT), one of the caffeic acid O-methyltransferase (COMT), and one cinnamoyl-CoA reductase (CCR). The final step before monolignol polymerization is mediated by peroxidases (PERs) and laccases (LACs). These two classes of enzymes catalyze the dehydrogenation of monolignols to produce radicals for the lignin-coupling reaction. The expression of DEGs related to most PERs and LACs was down-regulated in TZ compared SW-1 and SW-2, and only one PER-encoding and two LAC-encoding DEGs were up-regulated in TZ compared to SW-2, although they were in low expression levels (Figure 7; Table S6). This indicates that with the minimum or almost arrested activities of PER and LAC enzymes for channeling the monolignols into lignin biosynthesis, the remaining coniferyl alcohol in the cells could be supplied as the monolignol source for the subsequent lignan biosynthesis.

**Figure 8:**
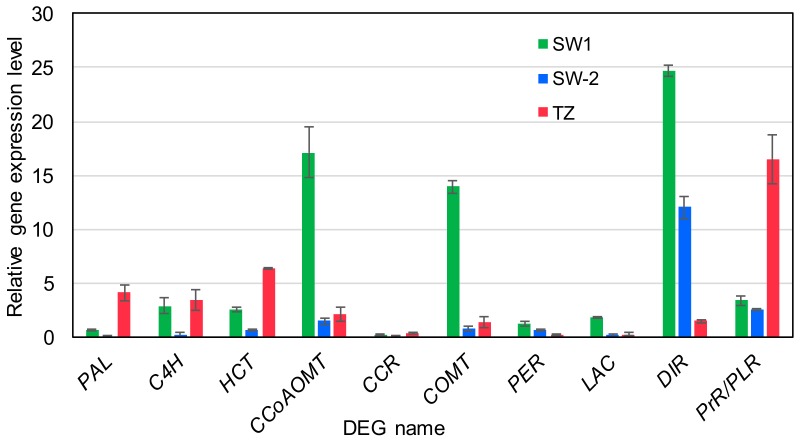
qRT-PCR validation of specific DEGs related to lignin and lignan biosynthetic pathway. PAL, phenylalanine ammonia-lyase; C4H, cinnamate 4-hydroxylase; HCT, 4-hydroxycinnamoyl-CoA shikimate/quinate 4-hydroxycinnamoyl transferase; CCoAOMT, caffeoyl-CoA O-methyltransferase; CCR, cinnamoyl-CoA reductase; COMT, caffeic acid O-methyltransferase; PER, peroxidase; LAC, laccase; DIR, dirigent protein; PrR/PLR, pinoresinol reductase/pinoresinol lariciresinol reductase. SW-1, outer sapwood; SW-2, inner sapwood; TZ, transition zone. Mean ± SD (n = 3).

With the aid of dirigent proteins (DIRs), two dehydrogenated coniferyl alcohols are radical coupled to form pinoresinol. Pinoresinol reductase (PrR) or pinoresinol lariciresinol reductase (PLR) further reduce pinoresinol into lariciresinol or secoisolariciresinol [33]. Finally, with the aid of other lignan-biosynthetic enzymes, other lignans, including taiwanin A, savinin, and helioxanthin, are biosynthesized. Only one DIR- and two PrR/PLR-encoding DEGs were up-regulated in TZ compared SW-2, and the expression levels of all other DIR- or PrR/PLR-encoding DEGs were down-regulated in TZ (Figure 7; Table S6). These three up-regulated DEGs are likely specific to the HW formation process, and provide the precursors for the subsequent lignan biosynthesis. Several DEGs from lignin and lignan biosynthesis pathways were randomly selected and further validated by qRT-PCR. The results (Figure 8) indicated that the expression patterns of selected genes validated from qRT-PCR generally match with the expression patterns from the transcriptome data, and the selected LAC and PrR/PLR genes did up-regulate in TZ compared SW-2 (Figure 8). These two genes, together with other monolignol biosynthesis-related DEGs expressed in TZ, might contribute to the gradual accumulation of the lignans in TZ of Taiwania stems during the HW formation process. In a transcriptome study of Scots pine (*P. sylvestris*) [27], researchers have shown that the elevated expression levels of PAL-, 4-coumarate CoA ligase (4CL)- and stilbene synthase-encoding transcripts led to stilbene biosynthesis in TZ and the accumulation of stilbene in HW. Similar results were also reported for eastern black walnut (*J. nigra*) and black locust (*R. pseudoacacia*), the accumulation of flavonoids in the TZ region were correlated to the elevated gene expression levels of chalcone synthase, flavanone-3-hydroxylase, and dihydroflavonol 4-reductase [22,25]. All these support the notion that biosynthesis of these phenolic compounds are activated in TZ during the HW formation process.

#### 2.4.3. Transcription Factors and Programmed Cell Death during HW Formation

**Figure 9:**
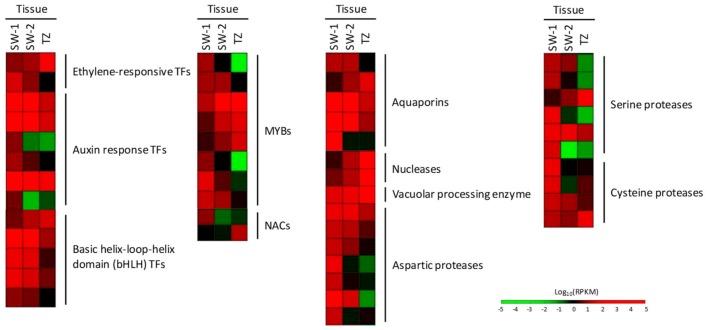
Expression profiles of DEGs related to transcription factors (TFs) and programmed cell death during the HW formation process in Taiwania. Each row represents the normalized gene expression levels of individual DEGs, and each column represents the expression levels from different tissues. SW-1, outer sapwood; SW-2, inner sapwood; TZ, transition zone.

It has been suggested that the plant hormones ethylene and auxin are involved in HW formation, and greater ethylene content and low or depleted auxin content have been reported to be in TZ [2,15,34]. Several transcripts related to hormone response transcription factors (TFs) were found in our DEGs (Figure 9), and they are ethylene-responsive and auxin response TFs. Most of these DEGs were down-regulated or in low expression levels in TZ, and only one ethylene-responsive TF was up-regulated more than 10-fold in TZ compared to SW-1 or SW-2 (Figure 9; Table S6). The function of this TF (ethylene response factor RAP, Table S6) is related to the regulation of gene expression by stress factors or stress signal transduction pathways, which might be related to the stress conditions resulting from the accumulation of massive secondary metabolites during HW formation in TZ. Research has shown that overexpression of the Arabidopsis ethylene response factor (RAP2.2) would result in improved plant survival under low-oxygen stress, whereas the T-DNA knockout lines had poor survival rates under the same stress [35]. There were five bHLH (basic helix-hoop-helix) TF-encoding transcripts found in our DEGs (Figure 9). In rice, bHLH TF ETERNAL TAPETUM 1 has proven to promote aspartic proteases triggering programmed cell death (PCD) in tapetal cells in rice anthers [36]. In these five DEGs, only one bHLH TF-encoding DEG was up-regulated more than 2-fold in TZ compared to SW-1 or SW-2, and all others were down-regulated (Figure 9; Table S6). Both positive and negative regulation of bHLH TFs might be related to the precise timing of HW formation induced PCD in TZ. Eight MYB- and two NAC-encoding transcripts were included in our DEGs, and three MYB- and one NAC-encoding DEGs were up-regulated more than 13-fold in TZ compared to SW-1 (Figure 9; Table S6). MYB transcription factors are often involved in secondary metabolism pathway regulation [37,38], and both transcription factor families are closely related to secondary cell wall biosynthesis and PCD [39,40]. However, the BlastX-searching results of these highly expressed MYB- or NAC-encoding DEGs in TZ did not reveal further specific functions related to wood formation associated TFs (Tables S5 and S6). Hence, it is worth taking a close look at these MYB and NAC TFs and their involvement and targeted genes in the HW formation process.

Four out of five aquaporin-encoding DEGs were down-regulated or in low expression levels in the TZ region compared to SW-1 or SW-2 (Figure 9; Table S6). Aquaporins facilitate the transport of water and small neutral solutes across membranes. Down-regulations of most aquaprins would unbalance the water homeostasis inside the ray parenchyma cells, cause dysfunctional or ruptured organelles inside the ray parenchyma cells, and might ultimately result in the cessation of water transport in the HW region. In the TZ of Japanese cedar, the water transport is precluded and it is within this zone that ray parenchyma cells die [41]. The earlywood of the intermediate wood (TZ) and HW of *L. kaempferi* also contained little water compared to the water-rich SW [42].

HW formation has been suggested as a special type of tissue senescence, and the PCD of ray parenchyma cells during HW formation shares some similarity to the PCD of xylem tracheary elements (TEs) [3,15,39]. In Taiwania’s TZ, ray parenchyma cells gradually lose their vitality (Figure 1b) and cellular contents [17]. DEGs encoding nucleases and vacuolar processing enzyme (VPE) were highly expressed in TZ with about 8- to 85-fold changes compared to SW-1 or SW-2 (Figure 9; Table S6). Both nucleases and VPEs have been reported as actively involved in regular plant PCD and TE differentiation [43,44], and the plant PCD process also harbors aspartic, serine, and cysteine protease activities in the autolytic process [43,45]. Aspartic protease-encoding transcripts were detected in our DEGs. However, most of them were down-regulated in TZ compared to SW-1 or SW-2, except one in TZ showing elevated expression levels (2.7-fold) compared to SW-2 (Figure 9; Table S6). This up-regulated aspartic protease-encoding DEG is likely associated with the up-regulated bHLH TF-encoding DEG involved in PCD during the HW formation process. However, the true association between these two genes awaits further investigation.

Among the serine protease-encoding DEGs, only one DEG related to serine protease was up-regulated 35- and 12-fold in TZ compared to SW-1 and SW-2, respectively (Figure 9; Table S6). This serine protease-encoding DEG was annotated as a serine carboxypeptidase-like gene. Research has showed that transcripts related to serine carboxypeptidase-like48 and some VPEs were up-regulated in plant organ senescence and many cell types that endured developmentally programed cell death (dPCD) [46]. This indicates that the heartwood formation in Taiwania might be closely related to developmentally controlled cell death. There are four cysteine protease-encoding DEGs. Two of them are annotated as cysteine endopeptidase (CEP), one of them is as xylem bark cysteine peptidase (XBCP), and one is as PPPDE thiol peptidase. Two DEGs related to CEP were up-regulated in TZ compared to SW-2 (Figure 9; Table S6). CEP has been identified as a key element of tapetal PCD [47], and is also considered as an indicator gene related to dPCD [46]. This further suggests that Taiwania’s HW formation is highly associated with dPCD. The expression level of XBCP gene gradually reduced from SW-1 and SW-2 toward TZ, indicating the down-regulation of this DEG in TZ, where the ray parenchyma cells began to die. Two closely related cysteine proteases, XCP1 (xylem cysteine protease 1) and XCP2, are considered key autolytic enzymes produced for the PCD of TEs [43], and these two genes were highly expressed before the cell death of vessel or fiber cells [48,49]. However, similar to the expression pattern of our XBCP gene, the expressions of these two XCP genes in the ray parenchyma cells of *Populus* showed decreases from outer SW toward inner SW, where the ray parenchyma cells gradually die [50]. This indicates that the cell death mechanism of ray parenchyma cells during the HW formation process might not fully resemble that in TE differentiation. The expression level of DEG predicted for PPPDE thiol peptidase increased more than 4- and 7-fold in TZ compared to SW-1 and SW-2, respectively (Figure 9; Table S6). The PPPDE thiol peptidase functions as a de-ubiquitinating and de-SUMOylating (small ubiquitin-like modifier, SUMO) peptidase, and is crucial for the regulation of cell cycles in many cellular processes, including protein stability, cell cycle progression, PCD, etc. [51,52]. This indicates that the ubiquitin signal network is also involved in the PCD of ray parenchyma cells during the HW formation process in Taiwania. Taken together, the cell death of ray parenchyma cells in Taiwania during the HW formation process harbors many dPCD-associated proteases and other lytic enzymes, and these different types of proteases and nucleases are closely involved in the clearance of the cellular contents of ray parenchyma cells in TZ.

## 3. Materials and Methods

### 3.1. Wood Tissues

Three well-grown Taiwania trees (about 35 year old and 22 m in height) were harvested in the late summer (September 2014) from Xitou Tract (23°40’10.8”N, 120°46’33.1”E; Elevation ~1358 m), in the experimental forest of the National Taiwan University at Nan-Tou County, Taiwan. All experiments were conducted using these tree trees (Tree A, B, and C). Increment cores were taken from breast height (1.3 m) of each of the Taiwania stems. Each increment core contained the characteristic orange-purple HW of Taiwania trees (Figure 1a). The increment cores were immediately submerged in a fixing solution containing 3.6% paraformaldehyde, 0.2% glutaraldehyde, 10% DMSO, 0.1% Nonidet P-40, 5 mM EGTA, and 5 mM MgSO_4_ in 50 mM PIPES buffer (pH 7.0) [53] for later living ray parenchyma cell profiling. Cross-section wood disks from where the increment cores were extracted were cut with a chainsaw on-site in the field, submerged in liquid nitrogen immediately, transferred back to laboratory in dry ice, and stored in a −80°C ultra-low freezer for later experiments.

### 3.2. Living Ray Parenchyma Cells and Major Bioactive Compound Profiling

The distribution of living ray parenchyma cells, total extractive content, and major bioactive compounds were profiled as per Chen et al. [17]. Briefly, the increment cores were separated ring by ring into different segments from cambium to HW, and these segments were cryostat-sectioned (Leica CM1900) at −20 °C to obtain the radial sections (40 μm in thickness). The radial sections were stained with Gill III hematoxylin and with 0.1% Safranin O, and were observed under an optical microscope (Olympus BX50). The percentage of living ray parenchyma cells in individual annual rings was calculated based on the number of ray parenchyma cells that contained nulei in a group of 100 ray parenchyma cells.

Tissues (about 500 mg) from the corresponding regions as indicated in Figure 1a (SW-1, SW-2, TZ, HW-1, and HW-2) were taken from the frozen cross-section wood disks. Each segment usually contained 2-3 annual rings, and spanned 1-3 annual rings away. These segments were pulverized under liquid N_2_ (IKA, A11), and extracted with 5 mL of methanol. The methanol extracts were rotavapor-dried and further dried over P_2_O_5_ under vaccum at room temperature. The percentage ratio of the vaccum-dried extractive weight over the vaccum-dried extractive-free woody tissue weight was defined as the total extractive content.

Aliquots (100 μL) from the methanol extracts above were mixed with the internal standard (caffeine, 4 μg) and profiled by high performance liquid chromatography (HPLC, Agilent 1200 series) equiped with a UV/Vis detector (280 nm) and a RP-18 column (Chromolith Performance RP-18e, 100 × 10 mm^2^ i.d., Merck). A gradient program using methanol (5–100%) and ddH_2_O (both contain 0.1% formic acid) was used to seperate the compounds at 25°C. Major bioactive compounds were authenticated as done by Chen et al. [17]. Relative peak area ratios from the bioactive compound peaks to the caffeine peak were used as the semi-quantitative comparison.

### 3.3. RNA Extraction and RNA-Seq

Tissues from the corresponding regions of SW-1, SW-2, and TZ of the frozen cross-section wood disks from 3 trees were used to isolate the total RNA according to a CTAB method [54]. Each of the frozen tissues were ground into fine powders in a freezer mill (IKA, A11) under liquid N_2_. The extraction buffer consisted of 2% CTAB, 2% PVP-40, 0.1 M Tris pH8.0, 25 mM EDTA, 2M NaCl, and 2% mercaptoethanol. RNA concentration and purity (A_260_/A_280_ and A_260_/A_230_) were measured with microvolume spectrophotometry (Nanodrop 2000, Thermo Scientific). A bioanalyzer (Agilent Technology) was used to evaluate the RNA qualities. Sequencing libraries were constructed by the Illumina TruSeq stranded RNA kit. Sequencing was performed by an Illumina NextSeq 500 sequencer provided by Genomics BioSci & Tech. (Taipei, Taiwan) to generate 150 bp paired-end reads. A total of 9 RNA-seq libraries, with 3 tissues (SW-1, SW-2, and TZ) per tree for 3 trees (Tree A, B, and C), were sequenced. The sequence data for these transcriptomes have been transferred to the National Center for Biotechnology Information (NCBI) under the SRA accession: PRJNA589955.

### 3.4. Transcriptome Assembly and Functional Annotation

Raw sequencing reads was trimmed to remove the sequencing adapters and the ambiguous reads having more than 45% bases with a Q-value < 20. Reads were then performed using sliding window trimming once the average Phred quality within a 4-base window fell below 20. Read trimming was performed by Trimmomatic [55] implemented in NTU Galaxy (National Taiwan University, Taipei, Taiwan). De novo transcriptome assembly was performed by Trinity [56,57] implemented in NTU Galaxy. Minimum assembled contig length to report was set as 200 bases. To reduce redundancy of assembled contigs, the longest transcript isoforms were retained as unigenes and were used as reference transcripts for gene differential expression analysis and functional annotation analysis. The unigene sequences were Blastx-searched against various databases, including NCBI non-redundant (nr), Swiss-Prot, and *Arabidopsis thaliana* protein sequences (TAIR 10.1) with an E-value threshold of 1 × 10^−5^. Gene ontology (GO) annotation was performed using the Blast2GO program (Version 4.1.9) [30].

### 3.5. Analysis of the Differentially Expressed Genes (DEGs)

Gene expression profiles and DEG analysis were conducted by CLC Genomics Workbench 7.0 (https://www.qiagenbioinformatics.com/). To compare changes in gene expression between three different tissues, we normalized gene expression levels to RPKM (reads per kilobase per million mapped reads) [58] by total mapped reads. Differential expression analysis was performed by the empirical analysis of the DGE tool in CLC that implements the edgeR exact test for two-group comparison [59,60]. Three pairwise comparisons were performed to identify DEGs: (1) SW-1 vs. SW-2; (2) SW-2 vs. TZ; and (3) SW-1 vs. TZ. The DEGs between tissues were further filtered for corrected FDR (false discovery rate) *p*-values < 0.005 and log_2_ (fold change) > 1. The FDR correction controls the expected proportion of incorrectly rejected null hypotheses and is used in multiple-hypothesis tests to reduce type-1 errors. Large-scale unigene and DEG differences between different tissues and biological replicates were analyzed by principle component analysis (PCA) using the multivariate method in JMP Pro13.0 (SAS Institute Inc., USA). Gene expression patterns between tissues and the heatmap for the filtered DEGs were generated by MATLAB R2017b (The MathWorks Inc. Natick, MA). GO term enrichment analysis was performed for the GO terms in DEGs, with the GO terms annotated from all unigenes as the reference, using Fisher’s exact test with multiple testing correction of FDR function implemented in the Blast2GO program (Version 4.1.9) [30]. General enriched GO terms were filtered from the result list at corrected FDR < 0.05. The DEGs were also mapped to the Kyoto Encyclopedia of Genes and Genomes pathway database (KEGG, http://www.genome.jp/kegg) [61].

### 3.6. Quantitative RT-PCR Validation of Selected DEGs

To validate the relative expression level of the selected DEGs in relation to certain biosynthetic pathways or functions, quantitative reverse transcription polymerase chain reaction (qRT-PCR) was performed. Total RNA was isolated from the corresponding tissues with a Plant total RNA Miniprep purification kit (Hopegen Biotechnology Development Enterprise, Taiwan). The cDNA was synthesized with a SuperScript^TM^ III first-strand synthesis system for RT-PCR (Invitrogen, USA). Quantitative reverse transcription PCR was conducted using a KAPA SYBR^®^ FAST qPCR Kit (KAPA Biosystems, USA). For each reaction, a 20 μl mixture containing < 20 ng template cDNA, 10 μL of 2× KAPA SYBR^®^ FAST qPCR Master Mix Universal (KAPA Biosystems, USA), and 200 nM each of the forward and reverse primer sets (Table S7). All reactions were performed in triplicate, and a melting curve of each sample was performed after the amplification program to confirm the single product amplification. The 18S ribosomal RNA gene of Taiwania was used as the internal control, and the relative expression levels were calculated based on comparative *C*_T_ methods [62].

## 4. Conclusions

The HW formation process of a Taiwania stem was analyzed. The extractive contents and major bioactive compounds gradually increased from the SW and TZ toward HW regions. Specific tissue regions (SW-1, SW-2, and TZ) that contain living or deceasing ray parenchyma cells were identified by observing the distributions of the ray parenchyma cells containing nuclei. The RNA-seq experiments assembled 36,654 unigenes, and the differential gene expression analysis could further retain 1428 DEGs representative to the HW formation process from the comparison of the three tissues. DEGs related to terpenoid biosynthesis pathways were all up-regulated in TZ to support the biosynthesis and accumulation of terpenoids. Many DEGs in lignin biosynthesis pathways were up-regulated, and two PrR/PLR-encoding DEGs were also up-regulated in TZ. These likely provide the precursors for subsequent lignan biosynthesis for the HW formation process. Several DEGs related to MYB and NAC TFs were up-regulated in TZ, and these TFs might be involved in secondary metabolite biosynthesis and PCD. Several nuclease- and protease-encoding DEGs were also highly expressed in TZ, and were likely involved in the autolysis of the cellular components of ray parenchyma cells in TZ during the HW formation process.

## Supplementary Materials

Supplementary materials can be found at https://www.mdpi.com/1422-0067/21/3/960/s1.

## Abbreviations

HW	Heartwood
SW	Sapwood
TZ	Transition zone
RNA-seq	RNA-sequencing
GO	Gene ontology
DEG	Differentially expressed gene
FDR	False discovery rate (*p*-value)
PCA	Principle component analysis
RPKM	Reads per kilobase per million mapped reads
MVA	Mevalonate
MEP	Methylerythritol 4-phosphate
IPP	Isopentenyl diphosphate
DMAPP	Dimethylallyl diphosphate
GGPS	Geranylgeranyl diphosphate synthase
TPS	Terpene synthase
FPS	Farnesyl diphosphate synthase
PAL	Phenylalanine ammonia-lyase
PER	Peroxidase
LAC	Laccase
DIR	Dirigent protein
PrR/PLR	Pinoresinol reductase/pinoresinol lariciresinol reductase
TF	Transcription factor
PCD	Programmed cell death
TE	Tracheary element
CEP	Cysteine endopeptidase
XBCP	Xylem bark cysteine peptidase
XCP	Xylem cysteine protease
EGTA	Ethylene glycol-bis(2-aminoethyl ether)-N,N,N’,N’-tetraacetic acid
PIPES	1,4-Piperazinediethanesulfonic acid
CTAB	Cetyltrimethylammonium bromide
