# Supplementary material for: Differential Gene Profiling of the Heartwood Formation Process in Taiwania cryptomerioides Hayata Xylem Tissues
Source: Int J Mol Sci. 2020 Jan 31;21(3):960. doi: 10.3390/ijms21030960 (PMC7037362; doi:10.3390/ijms21030960)
Supplement: Supplementary file 1 [file ijms-21-00960-s001.zip › Figure S1-S4.pdf]

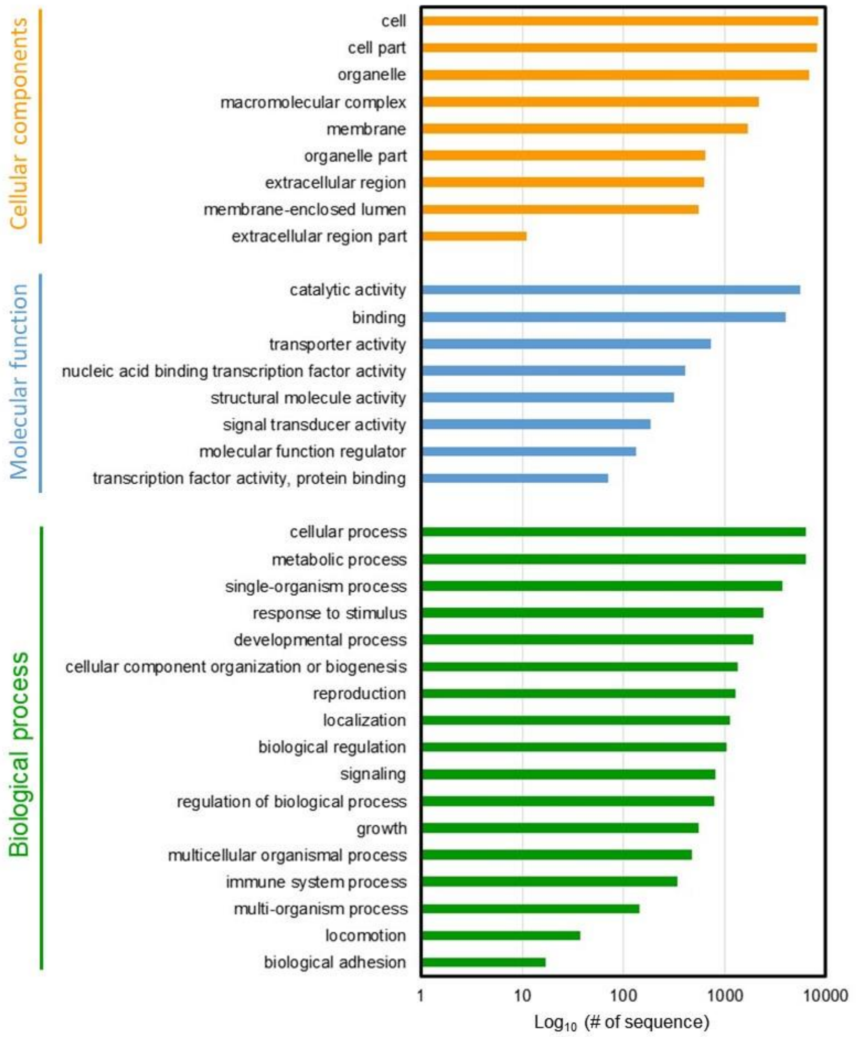

Figure S1. Functional annotation of the longest unigene collection based on gene ontology (GO) categorization.

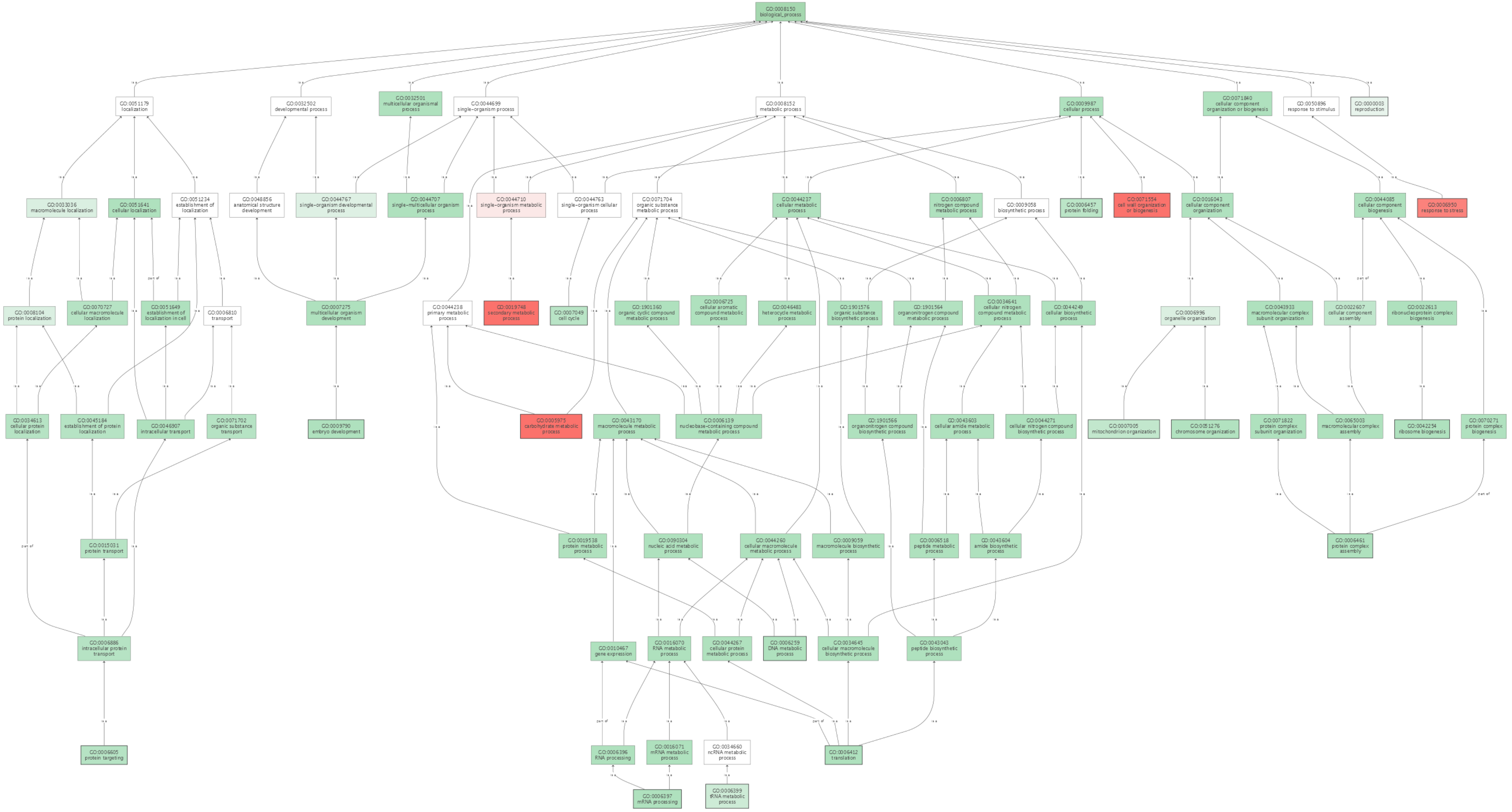

Figure S2. GO enrichment analysis for the DEGs in the "Biological Process" category. Red box indicates the enriched GO term in DEGs.

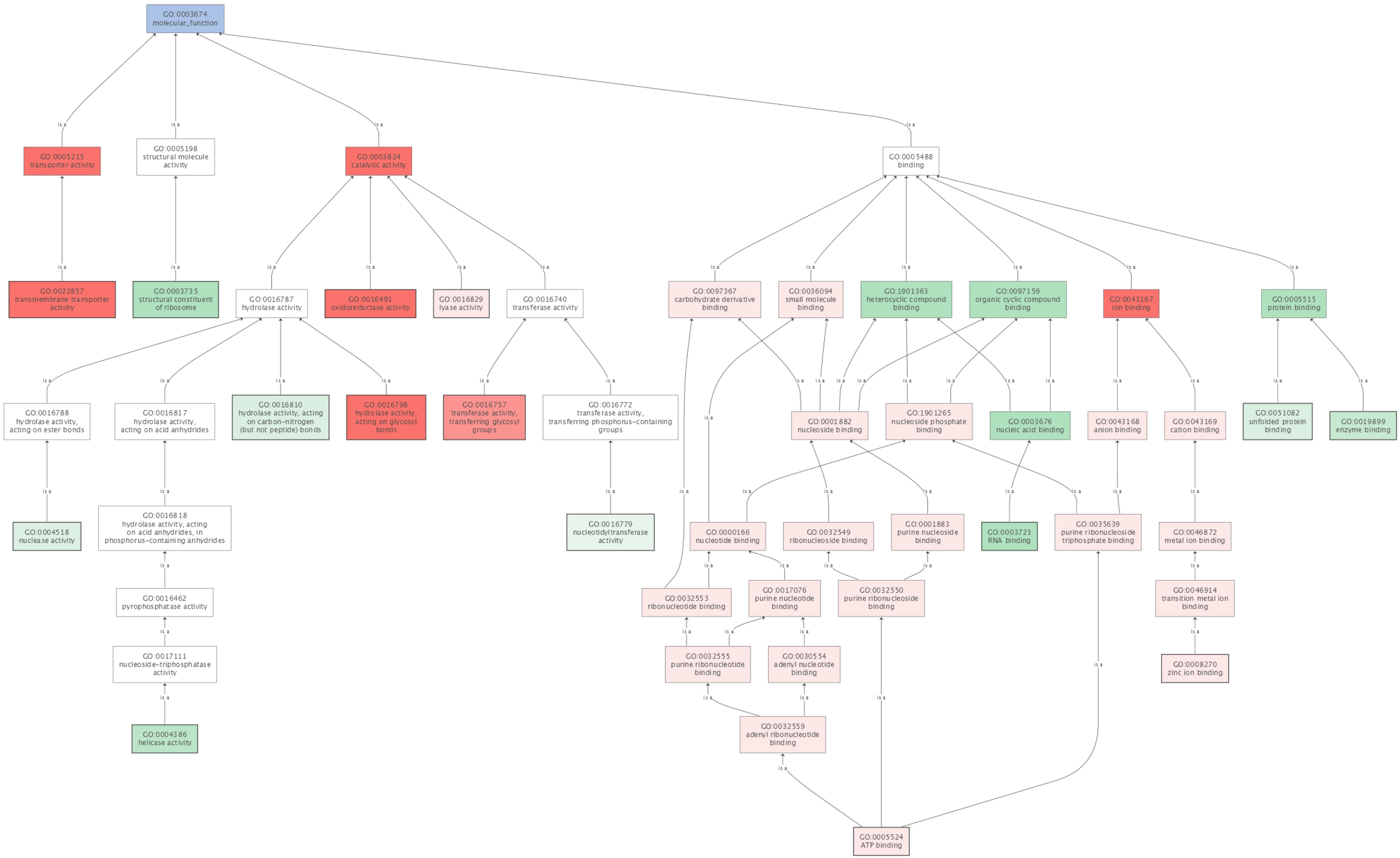

Figure S3. GO enrichment analysis for the DEGs in the "Molecular Function" category. Red box indicates the enriched GO term in DEGs.

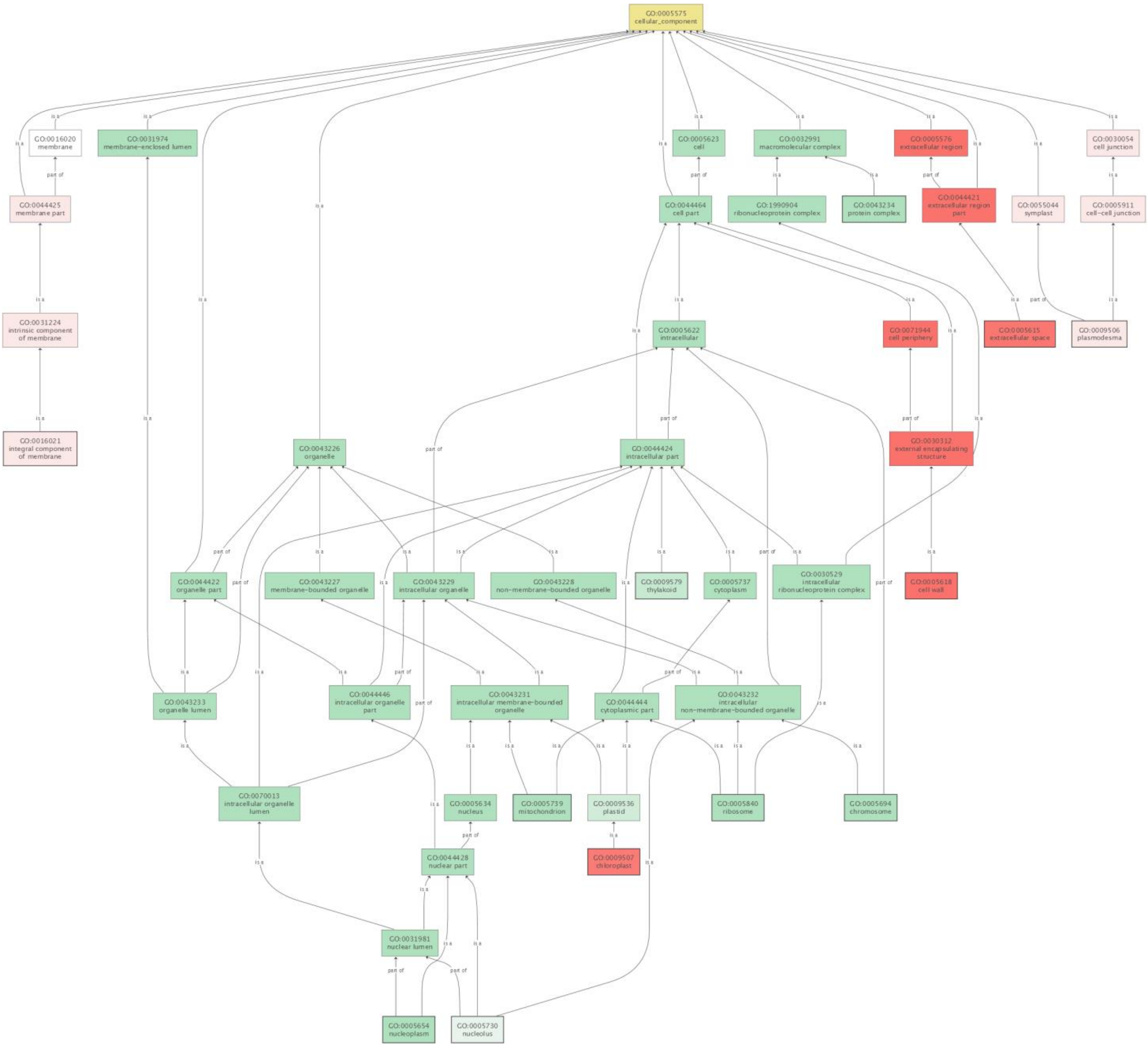

Figure S4. GO enrichment analysis for the DEGs in the “Cellular Component” category. Red box indicates the enriched GO term in DEGs.
